# Aortic Valve Sclerosis Adds to Prediction of Short-Term Mortality in Patients with Documented Coronary Atherosclerosis

**DOI:** 10.3390/jcm8081172

**Published:** 2019-08-05

**Authors:** Paolo Poggio, Laura Cavallotti, Veronika A. Myasoedova, Alice Bonomi, Paola Songia, Paola Gripari, Vincenza Valerio, Mauro Amato, Simone Barbieri, Pompilio Faggiano, Francesco Alamanni, Fabrizio Veglia, Mauro Pepi, Elena Tremoli, Damiano Baldassarre

**Affiliations:** 1 Centro Cardiologico Monzino IRCCS, 20138 Milan, Italy; 2 Dipartimento di Medicina Clinica e Chirurgia, Università degli Studi di Napoli Federico II, 80131 Napoli, Italy; 3 Cardiology Division, Spedali Civili and University of Brescia, 25122 Brescia, Italy; 4 Department of Medical Biotechnology and Translational Medicine, Università di Milano, 20129 Milan, Italy

**Keywords:** survival, surgical myocardial revascularization, EuroSCORE II, aortic valve sclerosis

## Abstract

Aims: Aortic valve sclerosis (AVSc), a non-uniform thickening of leaflets with an unrestricted opening, is characterized by inflammation, lipoprotein deposition, and matrix degradation. In the general population, AVSc predicts long-term cardiovascular mortality (+50%) even after adjustment for vascular risk factors and clinical atherosclerosis. We have hypothesized that AVSc is a risk-multiplier able to predict even short-term mortality. To address this issue, we retrospectively analyzed 90-day mortality of all patients who underwent isolated coronary artery bypass grafting (CABG) at Centro Cardiologico Monzino over a ten-year period (2006–2016). Methods: We analyzed 2246 patients and 90-day all-cause mortality was 1.5% (31 deaths). We selected only patients deceased from cardiac causes (*n* = 29) and compared to alive patients (*n* = 2215). A cardiologist classified the aortic valve as no-AVSc (*n* = 1352) or AVSc (*n* = 892). Cox linear regression and integrated discrimination improvement (IDI) analyses were used to evaluate AVSc in predicting 90-day mortality. Results: AVSc 90-day survival (97.6%) was lower than in no-AVSc (99.4%; *p* < 0.0001) with a hazard ratio (HR) of 4.0 (95%CI: 1.78, 9.05; *p* < 0.0001). The HR for AVSc, adjusted for propensity score, was 2.7 (95%CI: 1.17, 6.23; *p* = 0.02) and IDI statistics confirmed that AVSc significantly adds (*p* < 0.001) to the identification of high-risk patients than EuroSCORE II alone. Conclusion: Our data supports the hypothesis that a risk stratification strategy based on AVSc, added to ESII, may allow better recognition of patients at high-risk of short-term mortality after isolated surgical myocardial revascularization. Results from this study warrant further confirmation.

## 1. Introduction

Aortic valve sclerosis (AVSc) is a non-uniform thickening of valve leaflets with an unrestricted opening [1]. Based on the American Heart Association (AHA) and the American College of Cardiology (ACC) guidelines [2], AVSc does not compromise hemodynamics, having a maximal transvalvular velocity <2.5 m/s on Doppler echocardiographic measurement. This abnormality occurs in 30% of people who are older than 65 years of age and in more than 40% who are older than 75 years of age [3].

AVSc is characterized by inflammation, endothelial damage, lipid infiltration, and oxidation, with direct consequences on extracellular matrix degradation and calcification [4]. AVSc correlates with vascular risk factors (VRFs), subclinical atherosclerosis, and coronary artery disease and it is considered an atherosclerosis-like process [1,5]. In the general population, AVSc predicts ventricular hypertrophy and arrhythmias, myocardial infarction, stroke, and long-term cardiovascular (CV) mortality even after adjustment for VRFs and clinical atherosclerosis [6,7].

One of the models to estimate the risk of complications (e.g., mortality) after cardiac surgery is the EuroSCORE II (ESII); an algorithm that allows the evaluation of patient’s surgical candidacy and treatment selection [8]. In this study, we hypothesized that AVSc, in addition to ESII, behaves as a risk-multiplier, predicting mortality in patients with an overt atherosclerotic disease requiring coronary artery bypass grafting (CABG).

Thus, we have recorded 90-day mortality of consecutive patients who underwent isolated CABG at Centro Cardiologico Monzino, over a period of ten years, to assess whether AVSc is independently associated with CV mortality after isolated CABG.

## 2. Methods

### 2.1. Patients

This observational study was approved by the Institutional Review Board and by the Ethical Committee of Centro Cardiologico Monzino (CCM, University Hospital). The investigation conforms to the principles outlined in the Declaration of Helsinki (1964).

All patients who underwent isolated CABG from 2004 to 2015, at Centro Cardiologico Monzino, were included into this study. Demographic information, preoperative, intraoperative, and postoperative data were retrieved from the institutional database. The number of anastomosis, the on- or off-pump technique, as well as the graft used (e.g., saphenous vein, radial artery, and/or internal thoracic artery) were chosen by staff surgeons according to European guidelines [9].

**Figure 1:**
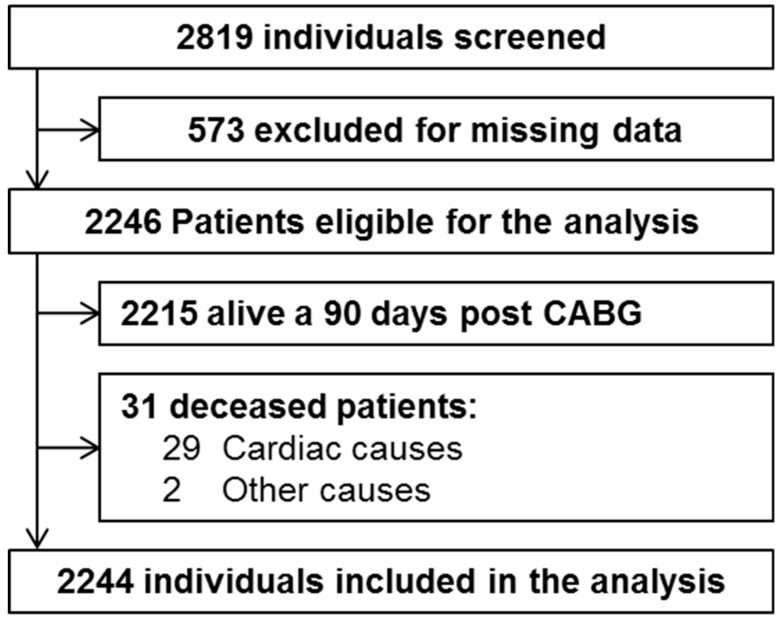
Case study selection.

A total of 2246 patients were identified. At the 90th day post-surgery, 2215 patients were still alive, whereas 31 were deceased (all-cause mortality: 1.5%). Deceased patients were eligible for the study only if the cause of death was cardiac (*n* = 29), their echocardiographic images were of good quality and they have no sign of significant valve pathologies (e.g., aortic valve stenosis, mitral regurgitation, or rheumatic heart disease). Therefore, the final analysis included 2244 patients. The flow chart with patient’s selection is shown in Figure 1.

### 2.2. Echocardiographic Evaluation

Patients referred to CABG undergo mandatory preoperative echocardiographic evaluation [9]. Experienced cardiologists of Centro Cardiologico Monzino performed the echocardiographic scans, according to the current guidelines [10,11]. The morphology and function of the aortic valve were assessed to evaluate the presence of AVSc. The AVSc was identified according to criteria described by Otto et al. [4]; i.e., non-uniform thickening or spotty calcified areas of the aortic valve leaflets without a significant transvalvular gradient (maximum aortic velocity <2.5 m/s). An expert cardiologist (P.G.) retrospectively evaluated the images blindly and, in case of uncertainty, another expert (M.P.) evaluated the echocardiographic scans. The agreement of the AVSc evaluation between the cardiologist who reassessed the images and the clinical records resulted in a weighted Cohen’s K = 0.88 (*p* < 0.0001).

### 2.3. Statistical Analysis

Quantitative variables with normal or skewed distribution were reported as mean ± SD or median and interquartile range, respectively. Categorical variables were reported as frequency and percentage. Group comparisons for normal, skewed, and categorical variables were performed by Student *t*-test for independent samples, by Wilcoxon rank-sum test, and χ^2^ test, respectively. The association between AVSc and 90-day mortality was assessed by Kaplan-Meier survival curves with log-rank test and by multivariable Cox regression analysis.

Three Cox models were implemented: (1) unadjusted; (2) adjusted for ESII; and (3) adjusted for a propensity score. The propensity score was computed, by logistic regression analysis, as the conditional probability of AVSc presence, given the following covariates: age, sex, body mass index, ejection fraction (EF), diabetes, dyslipidemia, hypertension, previous myocardial infarction, number of diseased coronaries, NYHA class, ESII, cardiopulmonary bypass (CPB), time on CPB, need of transfusion, and year of the intervention. In a sensitivity analysis, we also ran a Cox model adjusted for all the variables significantly associated with AVSc, even if the number of observed events would allow a maximum of two to three covariates in the model.

To assess whether AVSc adds to prediction of short-term mortality on top of ESII the integrated discriminating improvement (IDI) index was employed.

## 3. Results

The examined population was predominantly of male sex (84%) with an average age of 67 years and medium-low risk (ESII: 1.8 [95%CI: 1.1; 3.6]). Most patients (77%) had a three-vessel coronary disease with a mean left ventricular EF of 57%.

Among patients enrolled in the study who met the inclusion criteria (*n* = 2244), 1352 had normal aortic valve leaflets and were classified as no-AVSc and the remaining 892 (39.8%) had non-uniform thickening of the aortic leaflets and were classified as AVSc.

**Figure 2:**
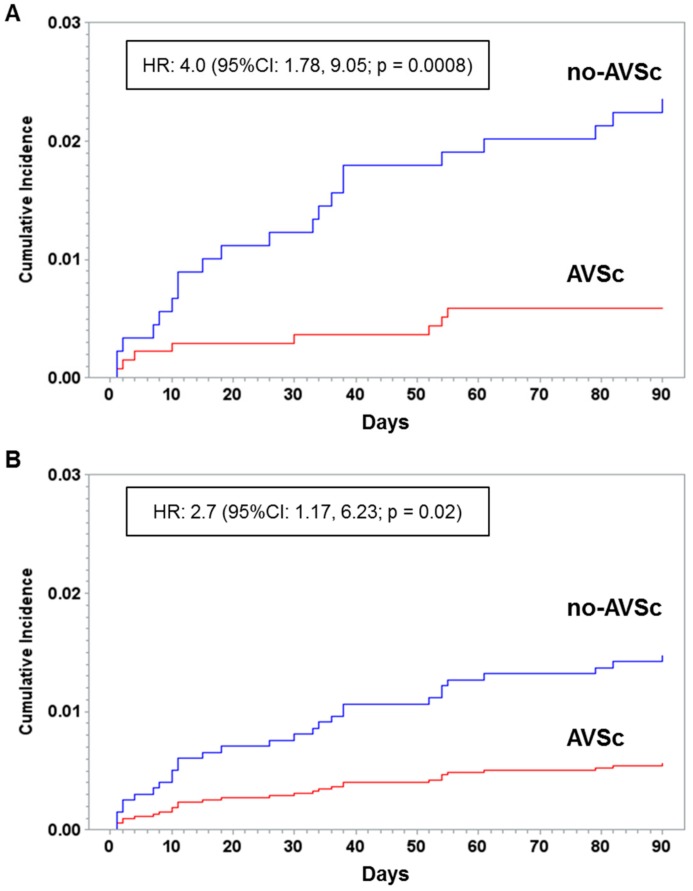
Kaplan-Meier cumulative incidence of patients that underwent surgical myocardial revascularization. (**A**) unadjusted and (**B**) adjusted for propensity score survival curves. Patients are stratified accordingly to normal aortic valve morphology (no-AVSc) or to aortic valve sclerosis (AVSc).

Table 1 shows the characteristics of patients with and without AVSc. The groups significantly differed for age, sex, prevalence of diabetes, NYHA class, EF, CBP and clamping time, and number of transfused units per patient. No differences were observed in terms of CAD severity, with the exception of the circumflex artery, which shows an increased number of patients with a stenosis >50% in the AVSc group. Kaplan-Meier cumulative incidence curves, stratified by presence/absence of AVSc, showed a significant increase in short-term mortality in AVSc patients (Figure 2).

After 90 days from CABG, the mortality rate in AVSc patients was 2.6% compared to 0.6% in no-AVSc patients. The unadjusted hazard ratio (HR) was 4.0 (95%CI: 1.78, 9.05; *p* = 0.0008), the ESII adjusted HR was 2.9 (95%CI: 1.26, 6.56; *p* = 0.01), while the propensity score adjusted HR was 2.7 (95%CI: 1.17, 6.23; *p* = 0.02). In the sensitivity analysis, the adjusted HR was minimally changed (HR: 2.44, 95%CI: 1.03, 5.77; *p* = 0.04).

In the IDI analysis, the model including AVSc and ESII performed better than the model including only ESII (IDI = 0.009, 95%CI: 0.004, 0.013; *p* < 0.001).

## 4. Discussion

In this study, we show for the first time that AVSc is an independent predictor of short-term (90-day) mortality after isolated surgical myocardial revascularization. The presence of AVSc, as identified in the routine screening before CABG, increases the risk of postoperative short-term mortality of more than two folds, and AVSc significantly adds to the identification of patients at high-risk for short-term mortality compared to EuroSCORE II alone.

Being adjusted for propensity score, our findings suggest: first, that the differences observed are not due to a simple unbalance of confounding factors in the two groups and second that AVSc probably reflects a pathophysiological mechanism that it is not fully explained by factors included in the current version of ESII.

To date, few studies have attempted to define the role of AVSc as a predictor of postoperative short-term and/or long-term outcomes. Cai et al. [12] reported a significant association between the presence of AVSc and post-operative major adverse cardiac events (which also included cardiac death) in 185 renal transplant recipients followed-up for a mean of 60 months; in such study, however, the short-term mortality was not considered. By contrast, in another study, the prognostic implications of AVSc on both post-operative short-term (30-day) and long-term composite outcomes were tested in 1172 patients with peripheral arterial disease requiring vascular surgery [13]. In multivariable regression analysis adjusted for age, gender, revised cardiac risk index, hypertension, hypercholesterolemia, and medication use, AVSc was not associated with either post-operative or long-term outcomes. According to these authors, the effect of AVSc was probably attenuated because their cohort was constituted by vascular surgery patients with extensive multi-vascular disease [13].

In the general population, AVSc is a rather common feature, with a prevalence ranging from 30 to 40% [1,3,13] and AVSc has been associated with an increased risk of all-cause and CV mortality in many [5,6,14,15,16,17,18,19] but not all [20] studies. Several authors have shown that the risk of dying from any-cause or for CV reasons in subjects with AVSc is significantly greater than in those without, even after adjustment for several risk factors [6,14,21]. Other authors, however, have shown that such increased-risk is totally eliminated when analyses were adjusted for CV risk factors [20]. AVSc, alone or included into cardiac calcium scores, also confers a greater risk of all-cause and CV mortality in subjects with no history of CV disease [14], in subjects with type 2 diabetes mellitus with or without history of myocardial infarction [16], and in patients hospitalized for overt or suspected CAD [22]. A recent meta-analysis reported a low (but still present) risk of all-cause and CV mortality in patients with AVSc [23]. Coffey et al. [18] hypothesized that the relatively high baseline risk of patients included in the study has masked the additional risk due to AVSc. This hypothesis, however, was contrasted by the results of the Cardiovascular Health Study, an observational cohort study of 3782 elderly patients followed up for 5 [6] or 6.6 [15] years, where AVSc remained an independent predictor of both all-cause and CV mortality regardless the presence/absence of baseline CAD. Our data, documenting an increased risk of mortality in patients who underwent CABG, adds another piece of information to this controversy.

Despite all the evidence reported in the literature documenting AVSc as a risk-multiplier, its capacity to act additively or synergistically to ESII for prediction of short-term mortality after CABG has never been tested. Our findings, showing (a) that AVSc predicts short-term CV mortality independently from ESII and (b) that AVSc adds to the identification of high-risk patients compared to ESII, strongly endorse AVSc as a promising factor to be integrated with the approaches currently used for the recognition of patients who are at high-risk of complications after isolated CABG.

### 4.1. Clinical Relevance

The identification of new risk factors for peri- and post-operative mortality is a core issue for guaranteeing high-quality results in risk-scoring systems. Several studies have reported a progressive worsening of the ESII model performances when applied to follow-up longer than 30 days [24]. In particular for isolated CABG surgeries, a poor calibration of ESII, in both the highest and lowest risk-patient groups, was reported [25]. To the best of our knowledge, our study is the first showing that AVSc could be combined with ESII to improve the identification of patients at high-risk of short-term mortality after CABG. Another point of relevance that deserves to be highlighted is that the detection of AVSc used for risk-prediction does not add any additional cost to usual clinical care, as the pre-operative echocardiography assessment is already mandatory in the evaluation of patients requiring cardiac surgery [9].

### 4.2. Strengths and Limitations

Keeping in mind its retrospective nature, our study has several strengths. First, is the tight control of the methodology for image acquisition and AVSc detection (all echocardiographic scans have been performed using the same protocol and all the scans were re-read blindly by a single reader with extensive experience). Second, the level of agreement of AVSc assessment among readers was strong [26] (Cohen’s Kappa = 0.88) even if derived from scans obtained from clinical practice. Finally, analyses were adjusted for operation related confounders (e.g., CBP time, transfusions, and year of intervention) that might have influenced the results. The study has also potential limitations. First, even if our analyses were performed on a large cohort, the number of events (i.e., mortality) recorded is small. Nevertheless, our results reach a statistically significant level. Second, our analysis was based only on a sample of CABG patients so, some caution must be applied before generalizing our results to other type of patients. Third, we could not distinguish CABG performed with or without CPB, due to the exiguity of the samples (dead patients with no CPB *n* = 1). However, the exclusion of the no CPB patient did not affect the results.

In conclusion, our data supports the hypothesis that a risk stratification strategy based on AVSc, added to ESII, may allow better recognition of patients at high-risk of short-term mortality after isolated surgical myocardial revascularization. Results from this study warrant further confirmation.

## Figures and Tables

**Table 1:** Demographic and clinical characteristics of patients deceased within 90 days from the intervention.

Variables	AVSc (*n* = 892)	No-AVSc (*n* = 1352)	*p*-Value
Age, years	69.4 ± 7.8	64.7 ± 9.1	<0.0001
Male *n*, (%)	708 (79.4)	1174 (86.8)	<0.0001
Body mass index, kg/m^2^	26.6 ± 3.5	26.8 ± 3.7	0.25
Diabetes mellitus *n*, (%)	298 (33.4)	367 (27.1)	0.0001
Hypertension *n*, (%)	668 (74.9)	989 (73.2)	0.36
Dyslipidemia *n*, (%)	628 (70.4)	1002 (74.1)	0.053
Current smoking *n*, (%)	147 (27.3)	246 (28.6)	0.84
NYHA heart failure class *n*, (%)			0.002
I	133 (15.7)	257 (19.9)	
II	523 (61.8)	811 (62.9)	
III	162 (19.2)	175 (13.6)	
IV	28 (3.3)	46 (3.6)	
CAD severity			0.23
1-Vessel coronary disease *n*, (%)	102 (11.4)	166 (12.3)	
2-Vessel coronary disease *n*, (%)	157 (17.6)	272 (20.1)	
3-Vessel coronary disease *n*, (%)	633 (71.0)	914 (67.6)	
Arteries with a stenosis ≥50%			
Long common trunk *n*, (%)	249 (27.9)	351 (26)	0.31
Anterior interventricular artery *n*, (%)	821 (92)	1234 (91.3)	0.52
Circumflex artery *n*, (%)	760 (85.2)	1104 (81.7)	0.03
Right coronary artery *n*, (%)	667 (74.8)	1006 (74.4)	0.84
Left ventricular ejection fraction, %	56.3 ± 10.6	57.9 ± 10.0	0.0008
Previous myocardial infarction *n*, (%)	364 (40.8)	506 (37.4)	0.11
CPB *n*, (%)	787 (88.2)	1219 (90.2)	0.15
CPB time, minutes	85.2 ± 41.4	90.8 ± 40.7	0.002
Clamping time, min	66.3 ± 22.6	70.3 ± 23.1	0.0002
Number of transfused units per patients, (interquartile range)	2 (0; 2)	0 (0; 2)	<0.0001
EuroSCORE II, (interquartile range)	1.6 (0.9; 3)	2.4 (1.3; 4.3)	<0.0001

AVSc, aortic valve sclerosis; NYHA, New York Heart Association; CAD, coronary artery disease; CPB, cardiopulmonary bypass.
